# Clarifying the Role of Urodynamics in the Preoperative Evaluation of Stress Urinary Incontinence

**DOI:** 10.1100/tsw.2008.159

**Published:** 2008-12-25

**Authors:** Sophie G. Fletcher, Gary E. Lemack

**Affiliations:** Department of Urology, University of Texas Southwestern at Dallas, 5323 Harry Hines Blvd., Dallas, TX 75390-9110, USA

**Keywords:** incontinence, urodynamics

## Abstract

It has not yet been definitively demonstrated that preoperative evaluation of women with stress urinary incontinence (SUI) with urodynamic testing (UDS) enhances surgical outcomes. Nonetheless, UDS is frequently utilized in the assessment of women with SUI in the hopes that results will shed light on preoperative risk factors for failure or postoperative voiding dysfunction. Poorer outcomes for stress incontinence surgery are primarily attributed to intrinsic sphincter deficiency (ISD), detrusor overactivity (DO), and voiding dysfunction. The ability of UDS to identify and characterize those parameters reliably remains under investigation. Furthermore, debate continues regarding the association of each of those factors with postoperative success for various SUI procedures. Since UDS is invasive, costly, and not always available, it is imperative that its benefit be carefully explored. In this review, we discuss the value of UDS in identifying risk factors for poor outcome and how those risk factors are associated with surgical failure.

## INTRODUCTION

Urodynamic testing (UDS) has clearly enhanced our understanding of bladder dysfunction among several different patient populations. For example, patients with neurogenic bladder conditions often benefit from testing in that more effective therapy can often be delivered when physicians have a more comprehensive understanding of bladder function. Additionally, patients at risk for upper tract deterioration due to severe neurovesical disease may be better identified through UDS. Although UDS has been utilized extensively to assess stress urinary incontinence (SUI) in women, its role in determining the severity of SUI and providing guidance for surgical planning for SUI remains speculative. Here we examine the urodynamic tools that have been developed to characterize SUI and summarize the available data that analyze the role of UDS in the preoperative setting.

## THE CLASSIFICATION OF STRESS URINARY INCONTINENCE

### Valsalva Leak Point Pressure

The valsalva leak point pressure (VLPP) was introduced by McGuire et al. as a urodynamic test to identify intrinsic sphincter deficiency (ISD)[1]. Defined as the lowest total bladder pressure at which urinary leakage occurs during progressive increases in intra-abdominal pressure, the VLPP was proposed as a measure of the mechanism and severity of SUI. McGuire reported that 76% of women with a VLPP ≤60 cm $H_2O$ had ISD (type III SUI). Urinary leakage due to bladder neck hypermobility (types I and II SUI) more commonly occurred with a VLPP >90 cm $H_2O$, and it has been suggested that grading the severity of SUI on the basis of VLPP might help with surgical planning[1]. However, at this point, using leak point pressure to evaluate patients with SUI and assist in surgical decision making is problematic, as the methodology of performing these measurements has not been standardized, its correlation with urethral closure pressure is poor, and its ability to predict surgical outcome has not been confirmed.

### *Measuring VLPP*

Various reports have documented discrepancy in pressure measurements according to catheter size, patient position, bladder volume, and provocative measure (cough vs. valsalva). Leak point pressure values can also vary greatly depending on whether total pressure or change in pressure from an abdominal pressure baseline is used[2]. Bump et al. demonstrated that VLPP measured with an 8F urethral catheter was significantly greater than with a 3F catheter[3]. Other investigators have confirmed this concept that larger catheters elevate VLPP, presumably via an obstructive effect[4]. Still others have suggested that flow rate can also be slightly impacted by the presence a urodynamic catheter, even among healthy volunteers[5].

Positioning of the patient is also critical to consider when carrying out leak point pressure measurements. Based on the original recommendation, the VLPP should be measured with the patient in the standing position. However, other authors have studied the parameter with the patient in a sitting position, as various clinical situations necessitate this. Siltberg et al. did not find a significant correlation between patient position and cough leak point pressures in 20 patients with genuine stress incontinence[6]. In a larger study looking specifically at this question, Nguyen et al. found that leak point pressures were significantly affected by the position in which the patients were tested. Less intravesical pressure was required to cause leakage of urine as the patient was progressively moved upright. Some patients did not leak in the supine position, but did leak in the semirecumbent and standing positions[7].

Further controversy surrounds the issue of bladder volume during measurement of VLPP. Several authors have reported the progressive lowering of VLPP as the bladder volume increased during filling in the same patients[8]. Petrou and Kollmorgen, however, found that bladder volume did not statistically change the VLPP determination[9]. A recent retrospective study also demonstrated that the bladder volume (100, 200, 300, 400, and >400 cc) at which women leaked during valsalva was not associated with any significant trend in VLPP[10]. Perhaps even more clinically relevant, these investigators found that leakage occurring at lower bladder volumes during VLPP was not associated with increased surgical failure after pubovaginal sling (PVS) or Burch colposuspension. There is currently no universally accepted volume at which VLPP should be performed. Finally, provocative measures used to measure VLPP produce varied results. Bump et al. reported statistically higher cough leak point pressure than VLPP[3]. In a prospective study by Sinha et al., all women with pure SUI leaked with cough during urodynamics, while only 34% of those women leaked with valsalva, as one might expect since the more rapid and robust change in intra-abdominal pressure with cough is more likely to cause SUI in the clinical setting[11]. If leak point pressure had been measured only via valsalva maneuver, 66% of patients with SUI could have been missed. Furthermore, these authors investigated VLPP in patients with the common clinical scenario of mixed urinary incontinence (MUI) and found that women with concomitant detrusor overactivity (DO) were more likely to leak with valsalva as the provocative maneuver. Multicenter studies using strictly applied urodynamic guidelines have elucidated the challenges in trying to standardize UDS between different clinical centers[12].

## Correlation with Symptom Severity

There have also been conflicting conclusions regarding the association of VLPP measurements to patient-reported symptom severity. A number of studies have demonstrated a correlation between leak point pressure and measures of incontinence severity including Stamey grade[1,13], SEAPI grade[2], incontinence episode frequency (IEF), and number of pads[3]. Conversely, others have published little to no correlation between VLPP and other measures of incontinence severity, such as pad weight and quality of life (QOL)[13,14], or the urogenital distress inventory (UDI) and the incontinence impact questionnaire (IIQ)[14]. The various methods for collecting symptom assessment and QOL data reported in these studies renders conclusive comparisons virtually impossible. However, recently, a multicenter randomized clinical trial of Burch colposuspension vs. PVS provided the opportunity to examine and compare prospectively collected standardized urodynamic measurements as well as measures of symptom severity[15]. The SISTEr trial, conducted by the Urinary Incontinence Treatment Network, randomized 655 women with pure or predominant stress incontinence to either Burch colposuspension or PVS. The primary outcome measure was surgical success of Burch vs. PVS. In a subsequent analysis, investigators examined the relationships between collected measures of incontinence severity (MESA [Medical, Epidemiological and Social Aspects of Aging] symptom questionnaire, UDI, IIQ, 3-day voiding diary, 24-h pad test, standardized volume stress test, and supine empty bladder stress test) and VLPP[15]. Additionally, a subset of patients with VLPP ≤60 cm $H_2O$ was classified as having ISD and was analyzed separately to determine whether severity measures correlated differently in patients with ISD. In the final analysis, no correlation was found between VLPP and any of the other incontinence severity measures. Furthermore, there was no improvement in correlation when a subgroup analysis of patients with VLPP above and below the traditional cutoff for ISD was examined[15]. Therefore, while VLPP may be a measure of urethral sphincter dysfunction, it does not appear to be associated with patient symptom severity, quantity of urine loss, or QOL measures.

## VLPP and Surgical Outcome

Beyond lack of systematic technique and poor correlation to symptoms, the use of leak point pressure to evaluate women with SUI has been ultimately problematic due to lack of surgical outcome data. As a determination of SUI severity and ISD, a lower VLPP on urodynamics has been used clinically to designate increased risk for surgical failure. Patients with type I or II SUI were traditionally thought to be better managed via retropubic suspension, transvaginal suspension, or anterior repair, while those with type III SUI were thought to be better managed via PVS, periurethral injection, or in the most severe cases, artificial urinary sphincter placement. However, subsequent studies have found this not to be the case and good outcome measures have been achieved in the case of all three SUI types (I, II, III)[16]. Until recently, these surgical outcomes were represented by a handful of observational studies with varying methodologies and outcome measures. However, a secondary aim of the multicenter randomized SISTEr trial, Nager et al. examined VLPP measurements and their prognostic value before Burch or PVS surgery[17]. Stratifying by treatment group, there was no difference in mean VLPP values between surgical successes and failures. Looking specifically at the VLPP cutoff of 90 cm $H_2O$, success rates were nearly the same (55 vs. 54%) for patients above and below the mark. From the above evidence, it appears as though VLPP does not confer any diagnostic advantage in women with uncomplicated SUI by history and physical examination.

As the tension free vaginal tape (TVT) and other midurethral synthetic sling (MUS) procedures are increasingly popular, the value of VLPP in predicting surgical outcomes beyond PVS and colposuspension is increasingly examined. Theoretically, MUS success is not predicted by sphincteric incompetence, but rather by urethral mobility, since they exert their effect in the mid- to distal urethra. The question arises then whether VLPP loses its predictive value in this setting. Several studies have shown that, as seen with the PVS, there is no apparent impact of VLPP on success following more distally placed synthetic slings. Rodriguez et al.[18] prospectively studied 174 patients and the impact of their preoperative UDS measurements on success following a distal polypropylene sling procedure for SUI. Patients were stratified by VLPP into four age- and symptom-matched groups: no leakage with urodynamics, VLPP <80 cm $H_2O$, VLPP 30–80 cm $H_2O$, and VLPP <30 cm $H_2O$. While patients with lower VLPP used more pads and reported more severe incontinence preoperatively, they fared no worse following surgery than those with higher VLPP or those who did not leak at all on UDS[18]. Further studies have compared TVT placement specifically in patients with ISD (VLPP <60 cm $H_2O$ and MUCP <20 cm $H_2O$) vs. those SUI patients without ISD (VLPP >60 cm $H_2O$ and MUCP >20 cm $H_2O$) and no statistically significant difference was seen in success rates with VLPP above and below the ISD threshold[19]. Success rates ranged from 74 to 94% at an average 12 months after surgery. Many have tried to assert mechanisms for success rates with TVT. High success rates have been attributed to an increase in pressure transmission to the urethra, but without a change in urethral closure pressure or decline in urethral hypermobility[20]. If this is the case, then VLPP measurements would not be expected to predict outcome, as the evidence above maintains.

Controversy does exist on the topic, however. This is to be expected whenever varied study designs and outcome measures are employed. Paick et al. found the overall cure rate at 10-month follow-up to be significantly lower in patients with lower VLPP (VLPP <60 cm $H_2O$/>60 cm $H_2O$ = 82:93%)[21]. O'Connor et al. found that the odds of persistent SUI following TVT-O were 12 times greater in patients with baseline VLPP ≤60 cm $H_2O$ compared to those with VLPP >60 cm $H_2O$[22]. A more recent study found significantly different cure rates in similar groups at a much longer 5-year follow-up interval after TVT (VLPP <60 cm $H_2O$/>60 cm $H_2O$ = 52:83%). Although on multivariate analysis, in this study, no independent risk factors were related to cure rate[23]. To date, there is still no conclusive Level 1 evidence to suggest that preoperative urodynamics predict outcome following TVT or other MUS procedures.

## Maximum Urethral Closure Pressure

While the VLPP is thought to measure the active component of the continence mechanism (e.g., the contraction of the sphincter with increase in bladder pressure due to abdominal strain), the maximum urethral closure pressure (MUCP) reflects the baseline tone of the sphincter. The technique of recording urethral pressure has been subject to significant variations, but in essence is an assessment of urethral function via the coaptive pressure of the urethral walls. This is measured using open or balloon-covered catheters perfused with liquid, or catheter-mounted (microtip) transducers. The catheter is withdrawn at a constant rate of speed and the pressure is measured from the bladder neck to meatus. As with VLPP, there is much debate regarding the technical reliability of urethral pressure profilometry (UPP). Many procedural variables contribute to artifact, which can significantly alter data: the type, stiffness, weight, and position (anterior vs. posterior wall) of the catheter; perfusion and withdrawal rates; patient position; and volume of bladder filling[24,25]. Furthermore, the reproducibility of MUCP is poor[26] and intraobserver variability is not well described.

Technical issues aside, the overall value of urethral profilometry as a UDS parameter for the identification and grading of SUI is unclear. While studies continue to demonstrate lower urethral pressures in women with stress incontinence as opposed to those without[27,28], overlap in MUCP values between patients with and without incontinence is ubiquitous. Thus, selecting a sensitive cutoff value for the diagnosis of incontinence is not feasible. Furthermore, despite multiple attempts identify a value of

MUCP that would characterize the severity of SUI, lack of standardization and variability in outcome measures limits conclusions. Although never independently validated, many associate ISD with values of MUCP ≤20 cm $H_2O$ and VLPP ≤60 cm $H_2O$[2,19,29].

## Correlation with Symptom Severity

As prognostic variables of SUI and potential surgical failure, VLPP and MUCP should be expected to correlate with measures of symptom severity. However, as seen with VLPP, there is no substantial evidence that MUCP correlates with reported symptoms. Bump et al.[3] sought to assess the accuracy with which the generally agreed criteria of MUCP ≤20 cm $H_2O$ and VLPP ≤60 cm $H_2O$ correlate to various severity measures of SUI, and recognized risk factors for ISD. No single parameter correlated very highly with either the diagnosis of ISD or even consistently with severity[3]. Subsequent authors have corroborated this, showing that no significant association has been demonstrated between pad test, bladder diary, QOL, and MUCP values[13,14,30]. Bump et al. suggested that no single factor be considered absolutely to establish the diagnosis of ISD, and only the combination of urodynamic measurements, patient history, and clinical severity should be used to guide management[3]. From the SISTEr trial, conclusive evidence exists to suggest that VLPP is not a corollary of incontinence severity measures[15]. As seen here, similar data for MUCP exist, although they are much less decisive. Furthermore, while some have demonstrated a modest correlation between VLPP and MUCP, and suggested that both are comparable in predicting incontinence severity, others have disproven that association. Many have demonstrated a substantial lack of association between low MUCP and low VLPP in women with SUI[3,30,31].

The question then arises whether MUCP is predictive of surgical success. In 1987, Sand et al. found that the group of women with urethral closure pressures ≤20 cm $H_2O$ had a 54% objective failure rate at 3-months follow-up, while an 18% failure rate was observed in the group with higher closure pressures[32]. Subsequent investigators also demonstrated that women with low urethral closure pressures were at increased risk of failure after Burch colposuspension[29,33]. For this reason, PVS emerged as the treatment of choice in the higher-risk group. However, Maher et al. found no significant difference in objective success rates between colposuspension and PVS in patients with low urethral pressures[34]. In a prospective randomized trial of 36 patients with an MUCP ≤20 cm $H_2O$, Sand et al. also reported no significantly different success rates between Burch and PVS[35]. Of course, variable methodologies and outcome measures make comparisons extremely difficult. Publication of results from the SISTEr trial provided evidence from a large, prospective randomized study comparing the surgical success of Burch colposuspension vs. PVS. However, MUCP was not evaluated as a preoperative risk factor. Well-powered evidence demonstrating the clinical value of MUCP measurements in predicting surgical outcome is eagerly anticipated.

TVT and other MUS procedures are designed to provide support at the midurethra, reinforcing the suburethral vaginal hammock as well as its connections to the pubococcygeus muscles. In other words, MUS placement corrects SUI by stabilizing urethral hypermobility. These procedures are not thought to influence urethral closure pressure, yet inconsistent reports on change in the degree of urethral hypermobility, urethrovesical angle, and MUCP in subjects after TVT confuse current understanding of this mechanism[36]. As seen earlier, there is considerable debate in the literature as to whether low VLPP predicts failure after TVT. However, while low urethral closure pressures (particularly MUCP ≤20 cm $H_2O$) have been associated with poor outcomes for conventional bladder neck operations, they do not appear to have similar predictive values for TVT. Liapis et al.[37] investigated 33 women undergoing TVT after a failed previous incontinence surgery. Success was independent of MUCP measurements and failure appeared to be most influenced by diminished mobility secondary to urethral fixation from scar formation[37]. Others have demonstrated success rates of 74–94% after TVT in patients with ISD[19,38]. Different definitions of ISD (MUCP ≤20 cm $H_2O$ alone vs. MUCP ≤20 cm $H_2O$ and VLPP ≤60 cm $H_2O$)

and varying criteria for success make comparisons difficult. Whatever the outcome measure, patients with ISD experienced the same favorable results as those without after TVT.

Varied success rates have been reported regarding transobturator tape (TOT) procedures in patients with low MUCP. Roumeguere et al. reported that subjects undergoing TOT with an MUCP ≤30 cm $H_2O$ had significantly lower success rates (76%) than those with higher urethral pressures (86%)[39]. Another study found equivalent results between groups, even with the threshold MUCP set lower at the more traditional ≤20 cm $H_2O$[40]. A more recent study examined combined VLPP and MUCP measurements for those values with the most sensitivity and specificity for failure[41]. A VLPP ≤60 cm $H_2O$ combined with an MUCP ≤40 cm $H_2O$ was the best model yielding a sensitivity of 83% and a specificity of 79%. The more traditional values used for ISD (VLPP ≤60 cm $H_2O$ and MUCP ≤20 cm $H_2O$) produced a sensitivity and specificity of 50 and 95%, respectively. In other words, these investigators experienced a meager 54% success rate with VLPP ≤60 cm $H_2O$ and MUCP ≤40 cm $H_2O$, and they recommend against using the transobturator sling for patients with SUI of that description. While these findings suggest that MUCP is useful to differentiate who will fail MUS procedures, more information from prospective trials randomizing patients to preoperative vs. no preoperative urethral pressure testing is needed to determine if surgical success can be predicted by the addition of this assessment.

## THE PREDICTIVE VALUE OF DETRUSOR OVERACTIVITY

MUI certainly poses a challenge when planning surgical management for a patient with SUI. Patient history can be difficult to interpret regarding the stress vs. urge components of the disorder. Measures of incontinence severity, such as QOL, voiding diaries, and pad tests, are not specific for urge incontinence and do not correlate well with the patients' reported symptoms[15]. While the simultaneous presence of DO may indicate MUI, controversy exists regarding the amplitude of DO and its predictive value[42]. Published reports have presented good data with sufficient follow-up showing that PVS[43], Burch colposuspension, and MUS[44,45] procedures are safe and effective in the setting of preoperative DO. In one study of women with both SUI and DO preoperatively, urodynamically proven resolution of DO was seen in 47% of patients after TVT[44]. Lee et al. found a 43 and 48% resolution of subjective urge and urge UI (UUI) in a large multicenter retrospective trial of TVT[23]. Recently, data from the SISTEr trial showed that DO was not predictive of failure in patients undergoing either PVS or Burch, although this conclusion may have been biased due to the selectivity for SUI-predominant patients in that trial[17]. Conversely, others have shown that when patients do fail MUS procedures, the finding of DO on preoperative UDS is the factor significantly associated[18,23,46]. As pointed out by Rodriguez et al., even in this patient population, treatment decisions based on UDS findings alone are difficult since a significant number of patients have resolution of UUI symptoms after surgery. Ultimately, further research needs to elucidate how varying degrees of DO predict serious postoperative complications vs. the identification of MUI that will respond well to PVS or MUS.

## PREDICTING POSTOPERATIVE VOIDING DYSFUNCTION

Voiding dysfunction may be the most concerning complication following anti-incontinence surgery. Although the reported incidence varies with the definition of voiding dysfunction used, up to 20% of patients will have new urinary complaints postoperatively[45,47]. Despite achieving incontinence cure rates of up to 90% (depending on the definition of success), only 70% of patients were subjectively satisfied with results after TVT. This is directly related to rates of new-onset urge symptoms[47]. While preoperative DO on UDS may account for cases of persistent urge and UUI after surgery, predictive factors of *de novo* urge symptoms are unknown. Gateau et al. found that predominant abdominal straining, as seen on UDS, was associated with *de novo* urgency after TVT[48]. This led to the hypothesis that postoperative *de novo* urge symptoms are associated with infravesical obstruction and that women with poor voiding pressures are unable produce an adequate flow rate against increased outlet resistance postoperatively. However, this premise still leaves many questions unanswered regarding variations in infravesical obstruction from patient to patient, and how UDS is predictive in this setting.

In contrast to *de novo* urgency, risk factors for voiding dysfunction after incontinence surgery have been elucidated. Age >65, additional surgical procedures, type of surgical procedure, postoperative cystitis, excessive elevation of the bladder neck, and menopausal status[49,50,51] have all been associated with varying degrees of impaired emptying in this setting. Regarding preoperative urodynamic evaluation in particular, patients with an absent detrusor contraction or valsalva voiding patterns were at risk of postoperative voiding dysfunction in this retrospective study. In a study of women who underwent PVS using allograft fascia, postoperative urinary retention (defined as inability to void 1 month after surgery) was seen in 5% of subjects[52]. All four of those patients were noted to void without a detrusor contraction (via urethral relaxation) on preoperative UDS, while patients who voided with a detrusor contraction had no difficulties voiding postoperatively. There were still 17 patients who voided without detrusor contraction on UDS and went on to have a normal postoperative course, making conclusions about the predictive value of this finding difficult. Wang et al. demonstrated that a Qmax <12 ml/sec and PdetQmax of ≥20 cm $H_2O$ were more likely to have a worse pad test result and lower QOL scores following TVT than those with normal pressure flow characteristics[53]. Others have demonstrated a significantly lower mean preoperative PdetQmax in patients with voiding dysfunction, as opposed to those without[38]. However, recent data from the SISTEr trial, which included women with SUI and at low risk for postoperative voiding dysfunction, showed that preoperative urodynamic parameters did not predict women likely to develop voiding dysfunction necessitating sling take-down[54].

The reason why TVT has been reported to cure DO, yet also is the cause of *de novo* urgency and voiding dysfunction, is unclear. The degree of sling tensioning, the position of the tape, and individual patient factors are all thought to play a role. Taken together, these studies imply that assessing pressure flow dynamics preoperatively can provide prognostic information regarding the development of voiding dysfunction. Patients who present with impaired emptying symptoms, elevated postvoid residual urine, or abnormal screening uroflowmetry may benefit from more in-depth UDS testing prior to surgery. However, there is no Level 1 evidence to suggest which patient populations may benefit most from urodynamics to clarify the risk of voiding dysfunction and, in fact, the largest prospective study on this topic suggests that, in a low-risk group, pressure flow studies did not predict patients likely to need intervention for persistent postoperative voiding dysfunction[54].

## CONCLUSION

UDS remains the most comprehensive tool in the evaluation of bladder and urethral function. Critical problems remain, however, regarding its role, especially in the preoperative evaluation of SUI. Among the most important of the problems are issues with standardization of the technique. It is not clear whether a comparison of UDS variables (VLPP, MUCP, DO) between centers is meaningful at this point. Furthermore, while conflicting data exist, there is no consensus that preoperative UDS improves patient selection or postoperative outcome for most patients. From recent multicenter randomized trials examining Burch and sling, evidence shows that UDS parameters traditionally thought to have predictive value (VLPP, DO) are not associated with surgical outcome. Finally, further research into individual patient factors, such as expectations before surgery, is necessary in order to counsel patients effectively in the preoperative setting.
